# Culture and Hybridization Experiments on an *Ulva* Clade Including the Qingdao Strain Blooming in the Yellow Sea

**DOI:** 10.1371/journal.pone.0019371

**Published:** 2011-05-05

**Authors:** Masanori Hiraoka, Kensuke Ichihara, Wenrong Zhu, Jiahai Ma, Satoshi Shimada

**Affiliations:** 1 Usa Marine Biological Institute, Kochi University, Usa, Tosa, Kochi, Japan; 2 Department of Natural History Sciences, Graduate School of Science, Hokkaido University, Kita-ku, Sapporo, Hokkaido, Japan; 3 Xianshan Xuwen Seaweed Development Co., Ltd., Jiangdong, Ningbo, China; 4 Shanghai Ocean University, Shanghai, China; 5 Division of the Natural/Applied Sciences, Graduate School of Humanities and Sciences, Ochanomizu University, Bunkyo-ku, Tokyo, Japan; Montreal Botanical Garden, Canada

## Abstract

In the summer of 2008, immediately prior to the Beijing Olympics, a massive green tide of the genus *Ulva* covered the Qingdao coast of the Yellow Sea in China. Based on molecular analyses using the nuclear encoded rDNA internal transcribed spacer (ITS) region, the Qingdao strains dominating the green tide were reported to be included in a single phylogenetic clade, currently regarded as a single species. On the other hand, our detailed phylogenetic analyses of the clade, using a higher resolution DNA marker, suggested that two genetically separate entities could be included within the clade. However, speciation within the *Ulva* clade has not yet been examined. We examined the occurrence of an intricate speciation within the clade, including the Qingdao strains, via combined studies of culture, hybridization and phylogenetic analysis. The two entities separated by our phylogenetic analyses of the clade were simply distinguished as *U. linza* and *U. prolifera* morphologically by the absence or presence of branches in cultured thalli. The inclusion of sexual strains and several asexual strains were found in each taxon. Hybridizations among the sexual strains also supported the separation by a partial gamete incompatibility. The sexually reproducing Qingdao strains crossed with *U. prolifera* without any reproductive boundary, but a complete reproductive isolation to *U. linza* occurred by gamete incompatibility. The results demonstrate that the *U. prolifera* group includes two types of sexual strains distinguishable by crossing affinity to *U. linza*. Species identification within the *Ulva* clade requires high resolution DNA markers and/or hybridization experiments and is not possible by reliance on the ITS markers alone.

## Introduction

**Figure 1:**
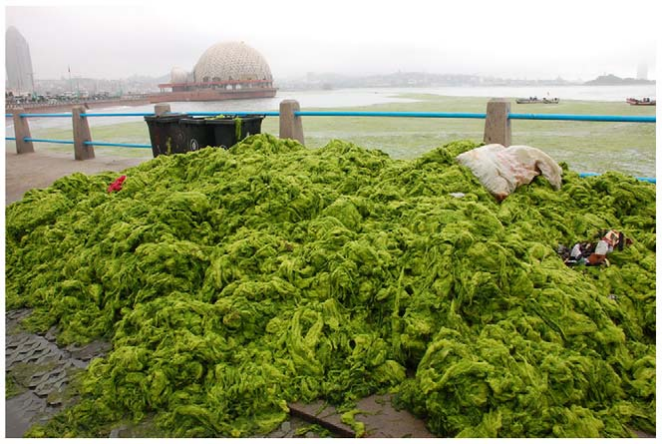
The collected *Ulva* blooming in the coastal waters of Qingdao, China on July, 2008.

Excessive growth of green macroalgae termed “green tides” have over the last four decades increasingly occurred in coastal areas worldwide, causing both ecological and economic impacts to coastal environments and human activities [1], [2]. In late June 2008, before the opening of the Beijing Olympics, a massive green tide of filamentous *Ulva* covering approximately 600 km^2^ along the coast of Qingdao, host city for the sailing regatta, lasted over two weeks, and over one million tons of the *Ulva* thalli were removed by more than 10,000 people (Fig. 1) [3]–[6]. As the cost to manage the impact of the 2008 Qingdao bloom is estimated as more than U.S. $100 million, proper management with efficient methods to suppress the *Ulva* growth is needed to prevent future massive blooms [6].

Different growth responses among various *Ulva* species to light, temperature, salinity and nutrient concentrations under controlled conditions have been reported [7], [8]. Different *Ulva* species bloom at different times and at different locations, so it is predicted that there are between-species differences of ecological impacts [9]. Recently, green tides composed of different species have been found to have different ecological functions, and are causing specific spatiotemporal occurrences [10], [11]. In order to understand the characteristics of green tides, a correct identification is important and it is an essential step to draw up an ecological profile of the species [4].

The genus *Ulva* presents extreme taxonomic difficulties, particularly in the types that form free floating blooms, because of their plasticity with season and environmental conditions and their broad intraspecific variations, whereas morphological differences between species are small [12]. Therefore, species identification in recent taxonomic studies of such green macroalgae has been confirmed using molecular markers such as the nuclear encoded rDNA internal transcribed spacer (ITS) region or plastid encoded large subunit ribulose 1,5-bisphosphate carboxylase gene (*rbc*L) [12]–[15]. Molecular analyses showed that the Qingdao strains are included in the *Ulva linza-procera-prolifera* clade (LPP clade) [4], [16], which is a monophyletic group on the basis of sequence analysis of the ITS region, including provisionally three *Ulva* species (*U. linza*, *U. prolifera* and *U. procera*) [17]. Taxonomically, the entire clade is currently regarded as *U. linza* [14]. However, in some publications the Qingdao strains are regarded as *U. prolifera* (or *Enteromorpha prolifera*) due to the filamentous, intensively branched morphology [18]–[21], because for *U. linza* normally a lack of branching is a diagnostic character [22], [23]. Furthermore, our detailed phylogenetic analyses of the LPP clade using a more resolved DNA marker, the 5S rDNA spacer, have suggested that two genetically separate entities could be included within the clade [17]. Although such taxonomic confusion in reference to the Qingdao strains occurs, it has not yet been examined whether the sequence-based species of the LPP clade contains two biological species or not. Thus, we conducted culture and hybridization experiments under controlled laboratory conditions in order to assess the species differentiation in genetic morphology and reproductive compatibility between the two entities within the LPP clade. Furthermore, the specific status of the Qingdao strains was evaluated and discussed.

## Materials and Methods

### *Ulva* strains

Collection data and characteristics of strains in the present study are listed in Table 1. Attached samples of *U. linza* and *U. prolifera* were collected from marine littoral habitats and brackish river mouths, respectively. Life histories of all strains of *U. linza* were firstly examined in the present study, while life histories of *U. prolifera* strains, except UPK, have been reported previously [24]. Collection of floating samples from the bloom was made at the third Bathing Beach, Qingdao, Shandong Province, China, where vast accumulations of filamentous *Ulva* thalli occurred in July, 2008 (Fig. 1). The floating samples were composed of numerous thallus fragments with dense branches, the morphology of which is consistent with that described in previous reports [19]–[21]. Two fragments were cultured for preliminary investigation. As they were indistinguishable in terms of developmental morphology of thallus branching and types of zoids, one of the two fragments was used for the crossing experiments.

**Table 1:** Origin of strains within the LPP clade used in this study.

					GenBank accession no.***	
Taxon*	Strain code	Life history (thallus type**)	Origin	Collection date	ITS	5S spacer
*U. linza*	ULC631	sexual (male gametophyte)	Oshoro, Otaru, Hokkaido, Japan	1-Mar-94	AB298633	AB298673
	ULC632	sexual (female gametophyte)	Oshoro, Otaru, Hokkaido, Japan	1-Mar-94	AB298633	AB298672
	ULO15	sexual (sporophyte)	Oshoro, Otaru, Hokkaido, Japan	7-May-07	AB298633	AB298673
	ULTM2	asexual by 4-flagellate zoids	Mugi, Kaifu, Tokushima, Japan	19-Feb-03	AB298633	AB298682
	ULS	asexual by 4-flagellate zoids	Shimoda, Shimanto, Kochi, Japan	17-Mar-06	AB298633	AB624457
	ULKM2	asexual by 4-flagellate zoids	Murotsu, Muroto, Kochi, Japan	4-Mar-03	AB299440	AB298683
	ULT	asexual by 4-flagellate zoids	None-chiku, Toyo, Kochi, Japan	23-Feb-04	AB624455	AB624458
	ULA	asexual by 4-flagellate zoids	Eshima, Awaji, Hyogo, Japan	29-Feb-04	AB624456	AB624459
*U. prolifera*	UPE21	sexual (sporophyte)	Shimanto Riv., Shimanto, Kochi, Japan	25-Feb-01	AB298320	c1:AB298658
						c2:AB298660
	UPK	sexual (sporophyte)	Koza Riv., Kushimoto, Wakayama, Japan	10-Feb-05	AB298316	AB624460
	UPE1	sexual (gametophyte)	Hiwasa Riv., Minami, Tokushima, Japan	29-Feb-00	AB298316	AB298665
	UPE20	asexual by 4-flagellate zoids	Yoshino Riv., Tokusima, Tokushima, Japan	7-Mar-00	AB298316	c1:AB298668
						c2:AB298665
	UPE3	asexual by 4-flagellate zoids	Niyodo Riv., Haruno, Kochi, Japan	7-Feb-00	AB298316	AB298665
	UPE8	asexual by 2-flagellate zoids	Hiwasa Riv., Minami, Tokushima, Japan	20-Apr-00	c1:AB298316	c1:AB298654
					c2:AB298319	c2:AB298655
	UPE19	asexual by 2-flagellate zoids	Yoshino Riv., Tokusima, Tokushima, Japan	23-May-01	AB298316	c1:AB298661
						c2:AB298665
	UPE2	asexual by 2-flagellate zoids	Niyodo Riv., Haruno, Kochi, Japan	7-Feb-00	AB298316	c1:AB298665
						c2:AB298666
Qingdao strain	QD	sexual (sporophyte)	No. 3 Bathing Beach, Qingdao, China	6-Jul-08	AB298314****	AB624461

*Determined by culture morphology of branch absence (*U. linza*) or presence (*U. prolifera*).

**Originally collected thallus type. In sporophytes, male and female gametophytes were cultured for hybridization.

***Accession numbers are recorded in the GenBank sequence database.

****This ITS ribotype is completely identical to ones from the other Qingdao strains previously reported [4], [19], [21].

### Culture and crossing experiments

The culture method and crossing experiments were described previously [24], [25]. Each strain was isolated by a unialgal technique using phototaxis of zoids. Life history of the strain was determined by microscopic observations of released zoids through at least two generations. When the original strain did not release gametes but zoospores, these could not be used directly for crossing tests. Therefore, the zoospores were cultured up to their gametophyte stage which can discharge gametes. Gametes from each strain were concentrated via positive phototaxis in autoclaved seawater. Small droplets of the dense gamete suspension from two different strains were mixed on a glass slide under a photomicroscope to observe any conjugation between them. As crossing ability of gametes swimming in autoclaved seawater became lower several hours after their release, crossing tests between different species or taxa were performed immediately after checking each for self-compatibility. Success or failure of copulation was confirmed by more than two replicate crossing tests. In successful copulation the hybrid zygotes that formed were isolated via negative phototaxis and cultured up to their reproductive stage, the sporophyte. Formation and release of zoospores in the sporophytes was also observed under a photomicroscope. Morphology of the cultured thalli of ≥20 mm long was observed to determine the absence or presence of branches.

### DNA phylogenetic analyses

DNA extraction, PCR amplification and automated sequencing were carried out for all strains, as described previously [17]. Sequences of ULO15, ULS, ULT, ULA and UPK, shown in Table 1, were newly determined since our previous reports [16], [17]. Phylogenetic analyses were performed using the maximum likelihood (ML) algorithm available in the computer program PAUP* 4.0 b10 with JC69 model [26]. Bootstrap values based on 100 re-samplings in ML of the dataset were calculated (TBR, full heuristic search option) [27].

## Results

**Figure 2:**
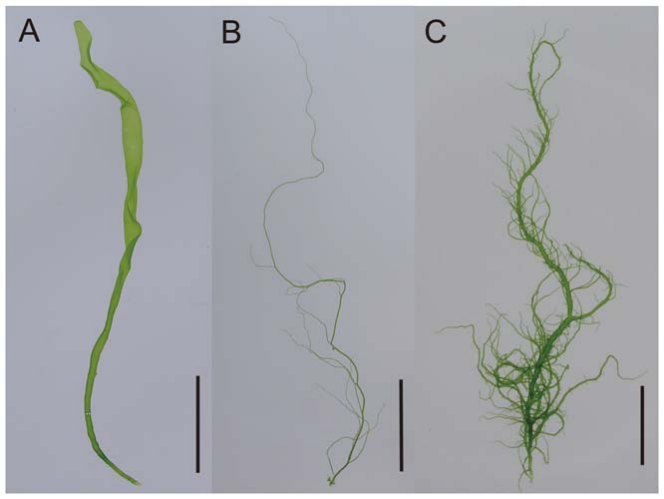
Cultured young *Ulva* thalli. (A) *Ulva linza* (ULC631). (B) *U. prolifera* (UPE21). (C) The Qingdao strain. The scale bars represent 5 µm.

**Figure 3:**
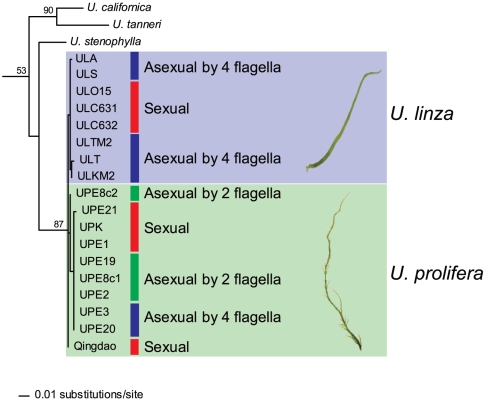
Phylogenetic tree of the ML analysis inferred from the ITS regions of the LPP clade. Initial letters UL and UP in the strain codes stand for *U. linza* and *U. prolifera*, respectively. Detailed information of each strain is given in Table 1. Numerals at internal nodes are bootstrap values >50% for 100 replicates in ML analysis.

**Figure 4:**
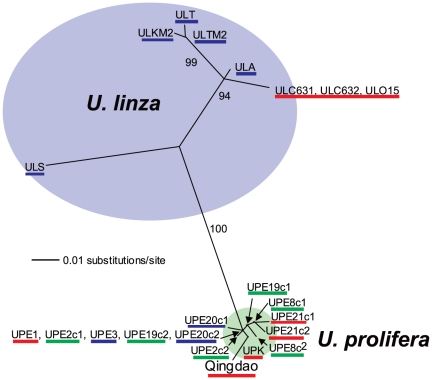
Unrooted maximum likelihood tree of the 5S rDNA spacer region of the LPP clade. Numerals at internal nodes are bootstrap values >50% for 100 replicates in ML analysis. As in Figure 3, red lines indicate a sexual type, blue lines an asexual quadriflagellate type and green lines an asexual biflagellate type.

The morphology and life histories of samples, determined by culture experiments, are shown in Table 1. Through several generations each strain repeatedly displayed the same type of thallus morphology in laboratory culture. Two morphotypes of the absence or presence of branches were distinguishable and taxonomically identified as *U. linza* and *U. prolifera*, respectively. The morphology of cultured thalli of the Qingdao strains was highly branching. The Qingdao strains had more branches than *U. prolifera* strains. Young cultured thalli had 4–6 branches per 1 mm in the Qingdao strains and 1–3 branches per 1 mm in *U. prolifera* strains (Fig. 2). The *U. prolifera* group included three different life histories being sexual, asexual with biflagellate zoids, and asexual with quadriflagellate zoids. Two filamentous thallus samples collected from the Qingdao bloom released the same types of quadriflagellate zoospores, which developed into gametophytes producing gametes. Therefore, it was determined that the Qingdao samples have a sexual life history with an alternation of isomorphic male and female gametophytes and sporophytes. In the *U. linza* group life histories of sexual and asexual with quadriflagellate zoids were found. The life histories of the LPP samples are placed on diagrams of ITS and 5S rDNA spacer regions (Figs. 3 and 4). Although division of the *U. linza* group and *U. prolifera* group was unclear on the ITS tree, the 5S rDNA spacer analyses clearly and consistently divided them into two groupings. Sexual and asexual strains intermingled within each group and did not fall into subgroups.

**Figure 5:**
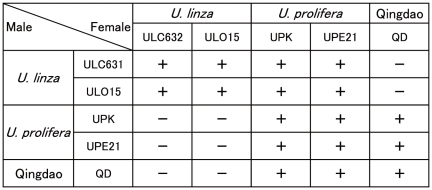
Hybridization matrix among *Ulva linza*, *U. prolifera* and the Qingdao strains. + = success of copulation, development of the isolated hybrid zygotes, and production of zoospores in the hybrid sporophytes, − = no observation of clumps or aggregations of mixed gametes.

Hybridizations were conducted among representatives of *U. prolifera* and *U. linza* and the Qingdao strains (Fig. 5). The results of *U. prolifera*×*U. linza* showed an unusual mating activity. Female gametes of *U. prolifera* successfully copulated with male gametes of *U. linza*, producing hybrid zygotes which can normally develop into sporophytes. In the successful copulation, after male and female gametes were mixed together, they immediately adhered to one another, the result being the formation of numerous clumps and zygotes by fusion of male and female gametes. However, in *U. prolifera* male×*U. linza* female no clumping occurred, indicating a complete gamete incompatibility in this combination. On the other hand, no sterility barrier between the Qingdao strains and *U. prolifera* strains was found in the laboratory experiments. Their hybrid zygotes normally developed into sporophytes which can produce zoospores. In the Qingdao strains×*U. linza* strains, no formation of clumps was observed in any combination of male/female gametes.

## Discussion

To suppress the considerable plasticity of *Ulva* thalli in response to changing environmental conditions, morphological characterizations of unialgal strains cultured under controlled laboratory conditions have been successfully conducted in previous taxonomic works of *Ulva* [22], [23], [28]. Also in the present study the culture experiments demonstrated the existence of at least two genetic morphotypes, *U. linza* and *U. prolifera*, within the LPP clade. Although the Qingdao strains with branches are categorized into *U. prolifera*, the degree of branches was denser than that of other strains of *U. prolifera*. The result suggests that the Qingdao strains may have different types of genes associated with branching patterns.

Only asexual strains have been previously reported in *U. linza* [23], [28]. As we first discovered sexual strains of *U. linza* in this study, crossing experiments could be carried out between *U. linza* and *U. prolifera*. The partial (asymmetric) reproductive isolation between *U. prolifera* and *U. linza* suggests a recent speciation of the two entities, that is consistently supported by the genetic separation, shown by an approximately 10 times more variable molecular marker, the 5S rDNA spacer, than the ITS region (Figs. 3 and 4) [17]. As contrasted with the asymmetric reproductive isolation between *U. prolifera* and *U. linza*, a complete reproductive barrier exists between the Qingdao strains and *U. linza* strains (Fig. 5). This implies that the *U. prolifera* group includes two types of sexual strains distinguishable by crossing affinity to *U. linza*. The results of hybridization experiments suggest that the Qingdao strains completed the speciation from *U. linza* via intermediate *U. prolifera*.

It has been reported that *U. intestinalis* widely occurs in the Baltic Sea with low salinities, while the distribution of *U. compressa* is restricted to salinities higher than 15 ppt and the limited distribution of the latter was explained by a lack of tolerance to low salinities [29]. Similarly, strains of *U. linza* are marine, not growing in oligohaline to mesohaline environments [23], [30], while those of *U. prolifera* are found commonly in estuaries and brackish waters [24], [30], having a wide range of salinity tolerance from oligohaline to euhaline conditions [22]. In our previous survey of *U. linza* and *U. prolifera* samples from a total of 53 populations from Japan, the two species had obviously distinct distributions, which are only marine seashore areas or mainly brackish river mouth areas, respectively [17]. Furthermore, the small proportion of *U. prolifera* that do inhabit marine seashores do not co-occur with *U. linza* [17]. Considering their habitat, it is thought that the salinity gradient may act as an isolating mechanism and cause the partial or complete reproductive isolation between *U. linza* and *U. prolifera* observed in our hybridizations.

Differences between *U. linza* and *U. prolifera* can be summarized as follows: (1) their genetic morphology was distinct by the absence or presence of branches, (2) they phylogenetically divide into two discrete clusters based on the 5S rDNA, (3) a partial or complete reproductive boundary exists between them, (4) they have different distributions. Thus, we conclude that *U. linza* and *U. prolifera* in the LPP clade should be regarded as two separate species. Although the Qingdao strains fall into *U. prolifera*, the different characteristics from the other brackish *U. prolifera* are recognized in the high degree of branches, the lack of crossing affinity to *U. linza*, and the marine habitat. Therefore, we consider that the Qingdao strains should be taxonomically dealt in infraspecific categories such as subspecies, varietas or forma. To establish the taxonomic status of the Qingdao strains, a more detailed comparison of diagnostic features, such as branching patterns, would be needed.

Sexual and asexual life history types have been reported in *U. prolifera* (as *Enteromorpha prolifera*) [24], [28]. In the present study we first found the Qingdao strains have the same sexual type of life history including an alternation of isomorphic sporophytes and male and female gametophytes, and a sexual mating process by male and female gametes. On the other hand, the asexual life histories reproducing by biflagellate or quadriflagellate zoids specialized for settlement have been cytologically proved to be quite independent and not to be the unusually prolonged phases of the sexual life history [24]. The asexual strains with quadriflagellate zoids of *U. prolifera* in our study can be taxonomically regarded as *U. procera* because *U. procera* has a branching morphology and an asexual life history by means of quadriflagellate zoids as key diagnostic characters [14], [22], [28]. Necessary nomenclatural changes resulting from this study will be formally addressed in a following paper.

Recently, ITS2, which is a part of the ITS region, has been suggested to be a molecular key to the level of biological species; in clades of eukaryotes, the identity of ITS2 predicts meaningful intercrossing ability, while a difference of even one compensatory base change in this region predicts total failure of crossing [31], [32]. Such a correlation of the molecular marker with reproductive barriers has also been predicted in *Ulva* taxonomy [33]. However, although all strains used in the present crossing experiments are identical in the ITS2 sequence, the crossing ability among them shows three different patterns which are successful crossing, partial or complete gamete incompatibility (Fig. 5). This high variation in mating ability within the LPP clade demonstrates that their mate recognition traits are evolving more rapidly than the ITS sequences. Many genes that mediate sexual reproduction, such as those involved in gamete recognition, diverge rapidly in eukaryotic organisms including the unicellular green alga *Chlamydomonas* [34], [35]. The mating process in *Ulva* is essentially similar to that in *Chlamydomonas* [36], [37]. In both algae, male and female gametes have two flagellae and the initial contact between mating gametes involves the flagellar tips of both mating types, which leads to the sexual agglutination and finally cell fusion [38]. Interestingly, female gametes of *U. prolifera* can copulate with both male gametes of *U. prolifera* and male gametes of *U. linza* and furthermore male gametes of *U. linza* can also copulate with both female gametes of *U. prolifera* and female gametes of *U. linza*. The crossing compatibility between these *Ulva* strains provides an important clue to the gamete recognition system, which directly correlates with speciation and reproductive isolation, particularly with pre-zygotic isolation. The molecular genetic evidence in *Ulva* must be detected on the model of *Chlamydomonas*, of which the mating system has been the most thoroughly studied [39].

Clades based on grouping procedures using ITS sequences are deductively considered to reflect genetically isolated entities, providing good resolution for identification at and below the species-level in a wide range of eukaryotic organisms [15] and also in *Ulva* [13], [14]. Therefore, tracking the algal origin of the green tide is being conducted by reliance on the ITS sequences [19]–[21]. However, we showed that a single clade based on the ITS sequences contains at least two separate biological species and their derivatively independent asexual strains. If the ITS markers are excessively relied upon as criteria to identify species, there is a risk of misunderstanding the ecophysiological characteristics of green tides by mistaking the actual causative strains.
